# CB_2_ Receptor Agonists Protect Human Dopaminergic Neurons against Damage from HIV-1 gp120

**DOI:** 10.1371/journal.pone.0077577

**Published:** 2013-10-17

**Authors:** Shuxian Hu, Wen S. Sheng, R. Bryan Rock

**Affiliations:** Center for Infectious Diseases and Microbiology Translational Research, Division of Infectious Diseases and International Medicine, Department of Medicine, University of Minnesota Medical School, Minneapolis, Minnesota, United States of America; Helmholtz Zentrum Muenchen - German Research Center for Environmental Health, Germany

## Abstract

Despite the therapeutic impact of anti-retroviral therapy, HIV-1-associated neurocognitive disorder (HAND) remains a serious threat to AIDS patients, and there currently remains no specific therapy for the neurological manifestations of HIV-1. Recent work suggests that the nigrostriatal dopaminergic area is a critical brain region for the neuronal dysfunction and death seen in HAND and that human dopaminergic neurons have a particular sensitivity to gp120-induced damage, manifested as reduced function (decreased dopamine uptake), morphological changes, and reduced viability. Synthetic cannabinoids inhibit HIV-1 expression in human microglia, suppress production of inflammatory mediators in human astrocytes, and there is substantial literature demonstrating the neuroprotective properties of cannabinoids in other neuropathogenic processes. Based on these data, experiments were designed to test the hypothesis that synthetic cannabinoids will protect dopaminergic neurons against the toxic effects of the HIV-1 protein gp120. Using a human mesencephalic neuronal/glial culture model, which contains dopaminergic neurons, microglia, and astrocytes, we were able to show that the CB_1_/CB_2_ agonist WIN55,212-2 blunts gp120-induced neuronal damage as measured by dopamine transporter function, apoptosis and lipid peroxidation; these actions were mediated principally by the CB_2_ receptor. Adding supplementary human microglia to our cultures enhances gp120-induced damage; WIN55,212-2 is able to alleviate this enhanced damage. Additionally, WIN55,212-2 inhibits gp120-induced superoxide production by purified human microglial cells, inhibits migration of human microglia towards supernatants generated from gp120-stimulated human mesencephalic neuronal/glial cultures and reduces chemokine and cytokine production from the human mesencephalic neuronal/glial cultures. These data suggest that synthetic cannabinoids are capable of protecting human dopaminergic neurons from gp120 in a variety of ways, acting principally through the CB_2_ receptors and microglia.

## Introduction

The global pandemic of HIV infection currently afflicts 34 million individuals, has killed over 25 million people since 1981, and is the cause of death in an estimated 1.8 million people per year [1]. Despite the therapeutic impact of anti-retroviral therapy (ART), HIV-1-associated neurocognitive disorder (HAND), which includes asymptomatic neurocognitive impairment, mild neurocognitive disorder, and the more severe HIV-associated dementia (HAD), still remains the most common form of dementia in adults under age 40 and remains an independent risk factor for mortality despite antiretroviral therapy [2]. As such, it remains a serious threat to AIDS patients [3-7] and there currently is no specific therapy for the neurological manifestations of HIV-1, in part because the underlying mechanisms mediating neurologic dysfunction remain poorly understood.

Several non-exclusive theories have been proposed as to why ART has not eliminated HAND [5], but among the most intriguing ideas is that even very low levels of viral replication in the central nervous system (CNS) could result in neural injury or dysfunction due to prolonged exposure to inflammatory responses and neurotoxic viral proteins [5]. In the setting of HIV in the CNS, the principal source of these inflammatory responses and neurotoxic viral proteins are microglial cells [8-11], the resident macrophages of the brain, which are the only brain cell types that can support productive HIV-1 infection. This idea is supported by the observation that clinical and pathological features of HAND correlate with macrophage/microglial cell activation rather than the quantity of infected cells or the viral load [12]. It is clear that in addition to being the key target of HIV-1 in the CNS, neurotoxic mediators released from activated microglia play a pivotal role in neuropathogenesis [13], including the HIV-1 envelope protein gp120 [14-20]. More specifically, HIV-1 gp120 appears to work both through direct toxic effects [21-23] and by interacting with microglial cells and macrophages, which then release a host of other neurotoxic mediators [24-27]. 

Looking at specific brain regions that are affected by HIV-1, others have demonstrated that the nigrostriatal dopaminergic system is a critical brain region for the neuronal dysfunction and death seen in HAD [28-38]. Dopamine (DA) is the main neurotransmitter in the nigrostriatal dopaminergic pathway and depletion of DA in this mesencephalic area underlies the clinical manifestations of Parkinson’s disease (PD). Clinical symptoms of PD have been well characterized in HAD [33,36]. Studies of simian immunodeficiency virus (SIV)-induced neuropathogenesis established that there is a major disruption within the nigrostriatal dopaminergic system, which is characterized by marked depletion of dopaminergic neurons, microglial cell activation, and reactive astrocytes [39,40]; these same histopathological abnormalities mirror those observed in HAD. In our laboratory, using a human mesencephalic neuronal/glial culture model, we were able to identify the relative sensitivity of dopaminergic neurons to gp120-induced damage, manifested as reduced function (decreased DA uptake), morphological changes, and reduced viability and that gp120-induced oxidative damage is involved in this neuropathogenic process [41]. Additionally, other studies show that dopaminergic neurons are reduced in patients with HAD [31,36], DA transporters are significantly reduced in HIV-1-infected patients compared with seronegative controls [38], and gp120 expression has been shown in the basal ganglia of patients with HAD [42]. 

Cannabinoids have been shown to alter immune cell functions [43], including certain functions of microglia, the resident macrophages of the brain parenchyma [44]. These activities appear to be mediated through cannabinoid receptors (CB_1_ or CB_2_). Synthetic cannabinoid agonists have been shown to induce beneficial effects in animal models of multiple sclerosis [45], Parkinson’s disease [46], and Huntington’s disease [47]. Work in our laboratory has demonstrated that certain synthetic cannabinoids inhibit the expression of HIV-1 in human microglial cells by a mechanism that involves CB_2_ receptors, as well as an atypical or non-CB_1_/CB_2_ receptor(s) [48,49]. Additionally, we have shown that activation of CB_1_ and CB_2_ receptors in human astrocytes suppresses production of inflammatory mediators [50,51]. To date, little is known about the neuroprotective effects of cannabinoids on human neurons, especially as these effects pertain specifically to neuroprotection against HIV-1.

Based upon findings that dopaminergic neurons are important to HIV neuropathogenesis, are preferentially sensitive to the effects of gp120, as well as the growing appreciation that oxidative stress plays an important role in HIV neuropathogenesis, experiments were performed using a human mesencephalic neuronal/glial culture model to assess whether the synthetic cannabinoid WIN55,212-2 ((R)-(+)-[2,3-dihydro-5-methyl-3-[(4-morpholinyl)-methyl]pyrrolo-[1,2,3-de]-1,4-benzoxazinyl]-(1-naphthalenyl)methanone mesylate) is able to protect against gp120-induced structural, functional, apoptotic, and oxidative damage to dopaminergic neurons, as well as examine the role that microglia play in this process.

## Results

Previously, we developed an *in vitro* human mesencephalic neuronal/glial culture model that contains approximately 55% neurons, 40% astrocytes, and 5% microglia. The neuronal composition includes 5% to 10% TH-immunereactive (IR) neurons (dopaminergic) relative to the neuronal cell population [41]. Our human mesencephalic neuronal/glial cultures do not contain noradrenergic cells and the relative amount of TH-IR neurons is comparable to what has been reported previously in human (2% to 5%) [52,53] and in rodent (1% to 2%) [54,55] mesencephalic neuronal/glial cultures. In this current study, a 3 hour pre-treatment model was used for all of the experiments involving cannabinoids. This paradigm was used based on our previous work performed evaluating the effect of cannabinoids on HIV-1 viral expression in human microglia [48,49]. The effect on viral suppression was similar when cells were pre-treated for 3 or 24 hours before infection, whereas cells exposed to WIN55,212-2 at the time of infection showed no effect on viral expression; this effect was stable out to at least 7 days [48]. Also, our previous work shows that exposure of our human mesencephalic neuronal/glial culture model to 10 nM of gp120 for 5 days imparted the most significant damage to dopaminergic neurons as measured by morphological changes, cell death, apoptosis, dopamine uptake, and lipid peroxidation [41]. Because of this, we used the same gp120 concentration and duration of exposure in all of the experiments below. Several other investigators have found that similar concentrations were necessary to elicit responses, including the use of gp120 in human microglia (100 nM) [14] and in rat neurons [10^-13^ M to 10^-8^ M [56], 2 nM [57] and 5 nM [58]]. Differences in the source of gp120 and the species used likely accounts for the diversity of concentrations used in these various publications. 

**Figure 1:**
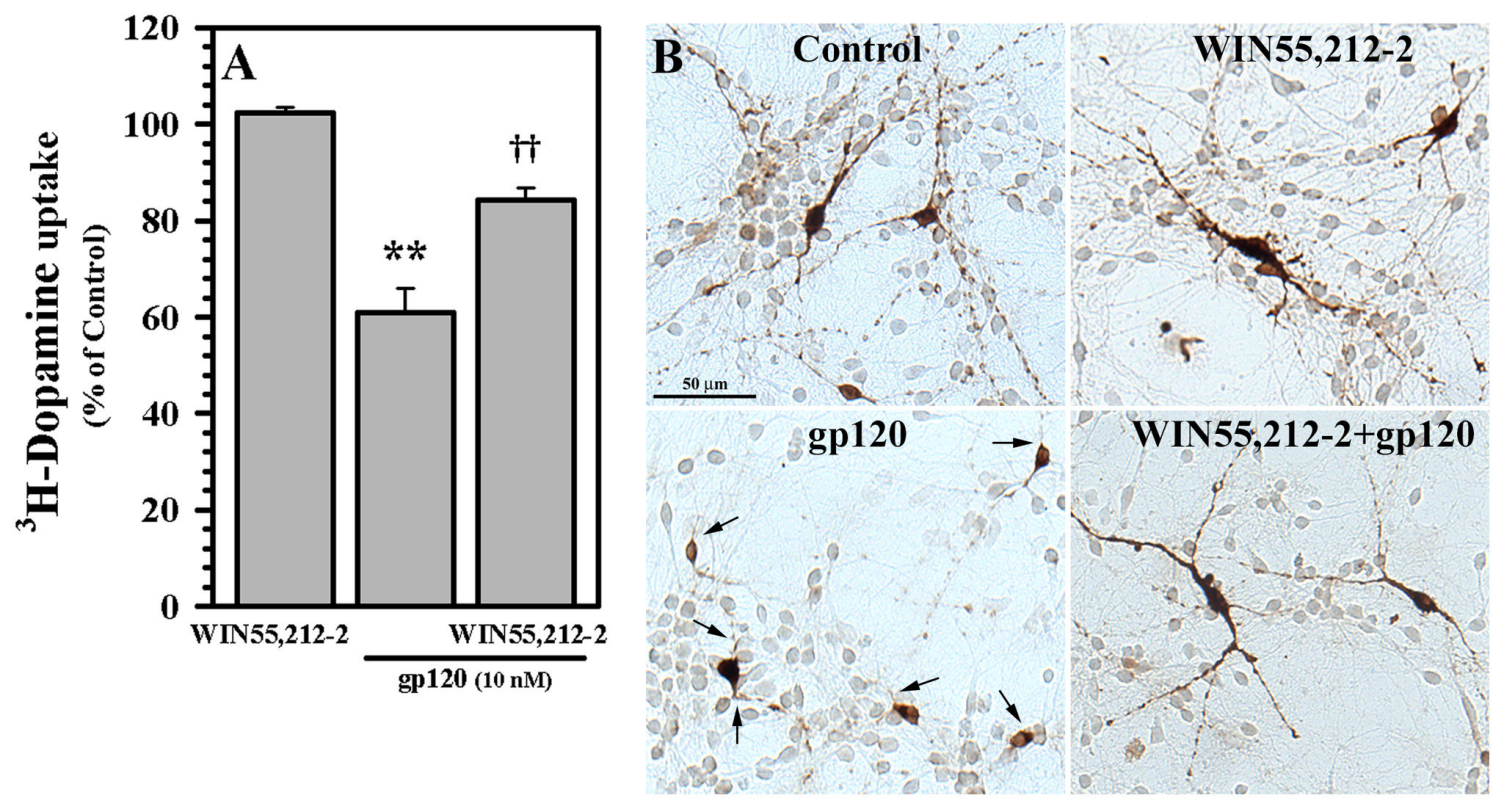
Blockade of gp120-induced dopaminergic neuron damage and suppression of dopamine transporter (DAT) activity by WIN55,212-2. **A**) Human mesencephalic neuronal/glial cultures were untreated (Control) or pretreated with WIN55,212-2 (0.3 μM) for 3h prior to gp120 (10 nM) treatment for 5 days followed by ^3^H-DA addition for 10 min as a measurement of ^3^H-DA uptake for DAT activity. **B**) Human mesencephalic neuronal/glial cultures were untreated (Control) or pretreated with WIN55,212-2 (0.3 μM) for 3h prior to gp120 (10 nM) treatment for 5 days followed by TH staining (arrows denote loss of dendrites). Data are mean ± SEM of triplicates and representative of 2-5 separate experiments using different brain tissue specimens. **p<0.01 vs. control; ††p<0.01 vs. gp120.

**Figure 2:**
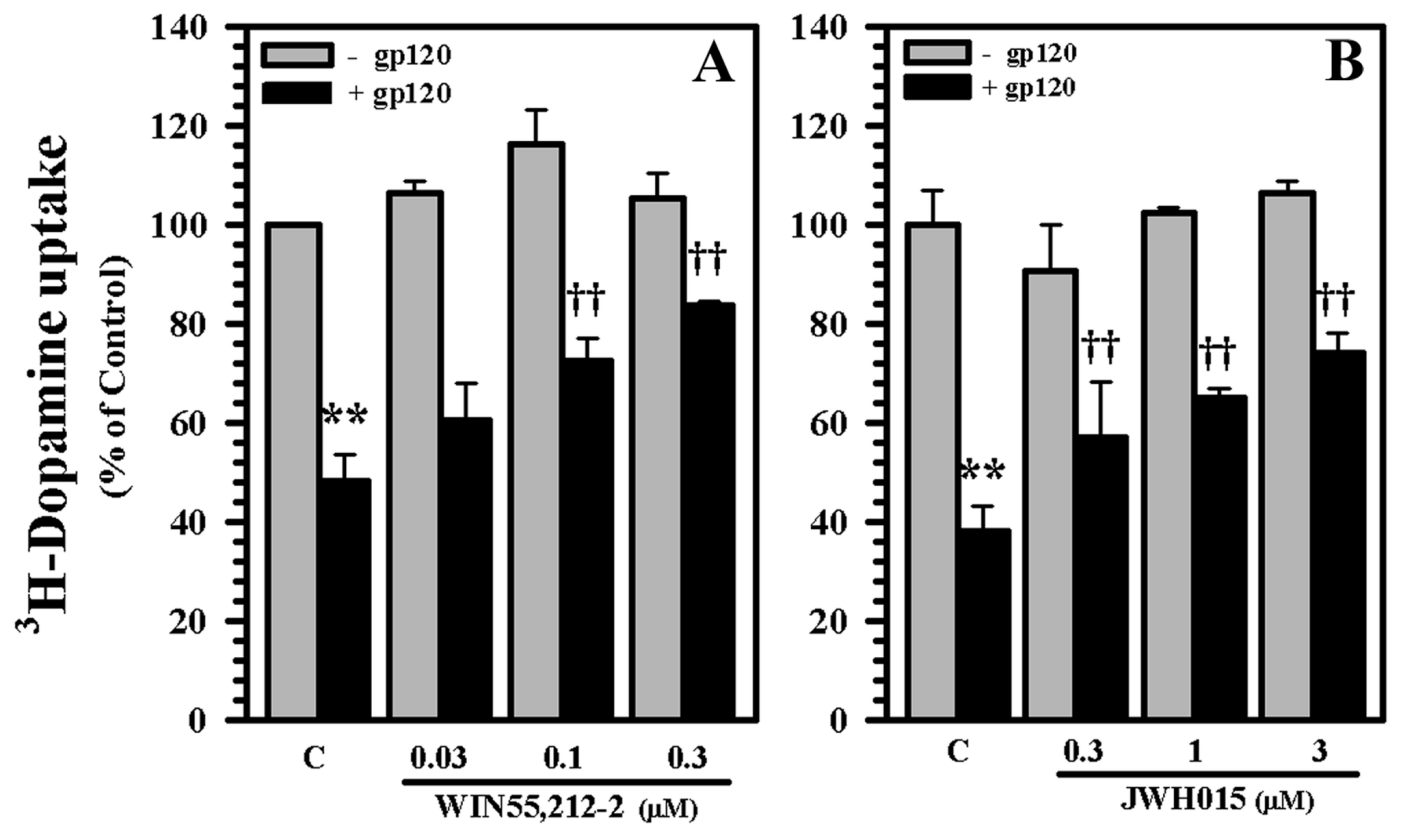
Blockade of gp120-induced suppression of DAT activity. Human mesencephalic neuronal/glial cultures were untreated (C) or pretreated with **A**) WIN55,212-2 or **B**) JWH015 at the stated concentrations for 3h prior to gp120 (10 nM) treatment for 5 days followed by ^3^H-DA addition for 10 min as a measurement of ^3^H-DA uptake for DAT activity. Data are mean ± SEM of triplicates of 3-4 separate experiments using different brain tissue specimens. **p<0.01 vs. control; ††p<0.01 vs. gp120.

Using our human mesencephalic neuronal/glial culture model, structural damage specifically to dopaminergic neurons was noted in response to gp120 in both loss of numbers and loss of dendrites (Figure 1B) to a degree similar to our previous work [41]. This process was avoided by pretreatment with WIN55,212-2, which is a CB_1_/CB_2_ receptor agonist (Figure 1B). In support of the notion that gp120 impairs DA uptake [41], we found that exposure to gp120 for 5 days decreased DA uptake (this effect was significant relative to heat-inactivated gp120 and anti-CXCR4 controls previously [41]). Treatment of these neuronal cultures with WIN55,212-2 significantly reduced (by 39.07 % ± 5.07%) this toxic effect of gp120 (Figure 1A). This protective effect of WIN55,212-2 was dose dependent (Figure 2A). The use of the CB_2_ agonist JWH015 also showed a trend toward a dose dependent protective effect as well (Figure 2B), suggesting the importance of the CB_2_ receptor.

**Figure 3:**
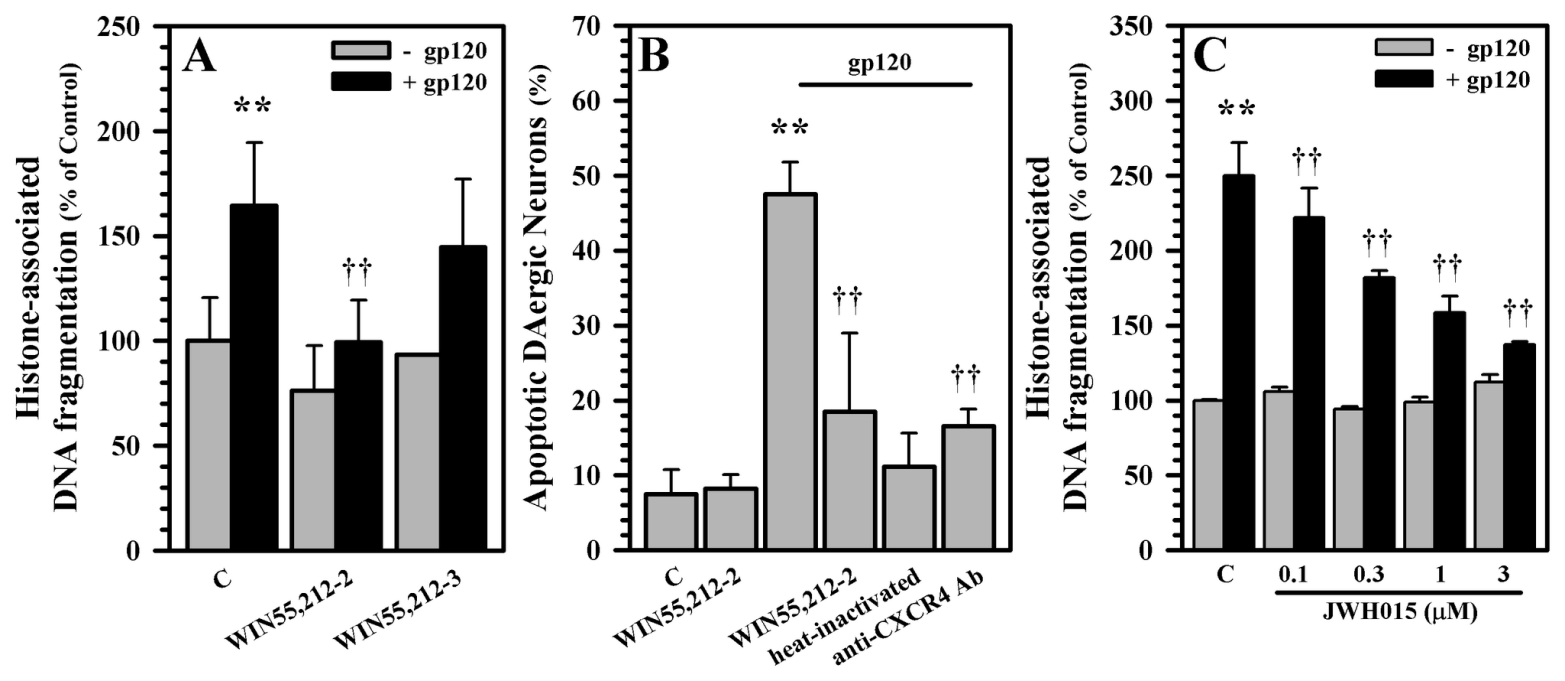
Blockade of gp120-induced apoptosis of dopaminergic neurons in human mesencephalic neuronal/glial cultures. **A**) Human mesencephalic neuronal/glial cultures were left untreated (C) or pretreated with WIN55,212-2 (0.3 μM) or the inactive enantiomer WIN55,212-3 (0.3 μM) for 3h prior to gp120 (10 nM) treatment for 5 days; apoptosis was measured by cell death ELISA. **B**) Human mesencephalic neuronal/glial cultures were either untreated (C), pretreated with WIN55,212-2 (0.3 μM) for 3h or anti-CXCR4 Ab for 1h prior to gp120 (10 nM) treatment or treated with heat-inactivated gp120 for 5 days. After fixation and permeabilization, dopaminergic neurons were identified by immunostaining for TH and apoptotic cells were assessed by cell counting with propidium iodide staining. **C**) Human mesencephalic neuronal/glial cultures were untreated (C) or pretreated with JWH015 (0.1 to 3 µM) for 3h prior to gp120 (10 nM) treatment for 5 days; apoptosis was measured by cell death ELISA. Data are mean ± SD of triplicates and representative of 2-4 separate experiments using different brain tissue specimens. **p<0.01 vs. control (C); ††p<0.01 vs. gp120; p< 0.05 between (WIN55,212-2+gp120) vs. (WIN55,212-3+gp120) (in A).

Because neuronal apoptosis is one of the histopathological hallmarks of HAD, we exposed our cultures to gp120 and assessed the level of neuronal apoptosis. Supporting our hypothesis, gp120 treatment induced apoptosis in our human mesencephalic neuronal/glial cultures (Figure 3A), and WIN55,212-2 significantly blunted both global apoptosis in this model (Figure 3A), as well as dampened dopaminergic neuronal apoptosis specifically (Figure 3B); heat-inactivated gp120 did not enhance neuronal apoptosis and anti-CXCR4 antibody appropriately blocked the gp120 effect. The use of the CB_2_ agonist JWH015 also showed a dose dependent protective effect as well (Figure 3C), again suggesting the importance of the CB_2_ receptor.

**Figure 4:**
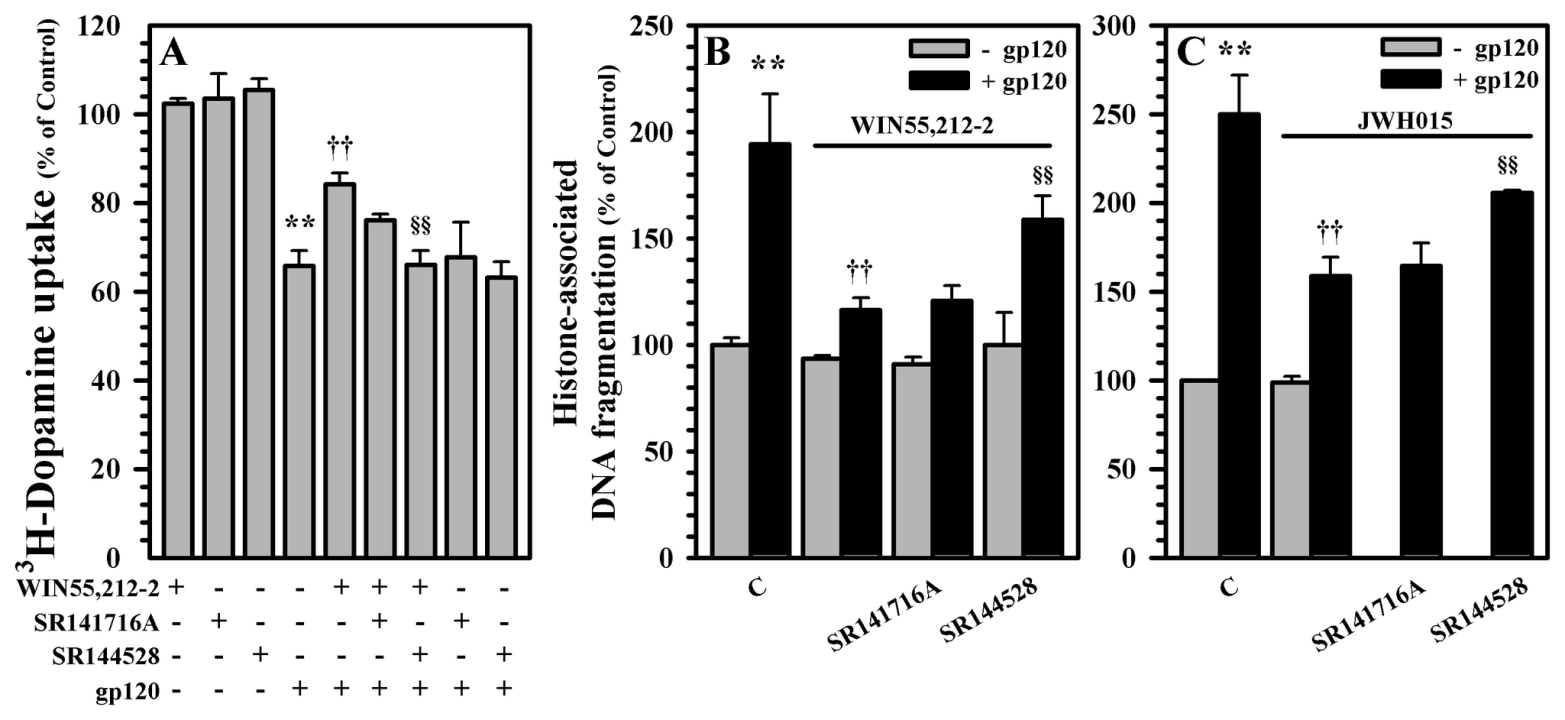
Involvement of cannabinoid receptors in gp120-induced suppression of DA uptake and apoptosis. Human mesencephalic neuronal/glial cultures were untreated (C) or pretreated with SR141716A (CB_1_ antagonist) or SR144528 (CB_2_ antagonist) (1 μM) for 30 min prior to WIN55,212-2 (0.3 μM) or JWH015 (CB_2_ agonist) (1 μM) addition for 3h followed by gp120 (10 nM) treatment for 5 days to measure **A**) ^3^H-DA uptake and **B**, **C**) apoptosis by cell death ELISA. Data are mean ± SEM of triplicates of 2-4 separate experiments using different brain tissue specimens. **p<0.01 vs. control; ††p<0.01 vs. gp120; §§p<0.01 vs. WIN55,212-2+gp120 (in A, B) or JWH015+gp120 (in C); p<0.05 between (SR144528+JWH015+gp120) vs. (SR141716A+JWH015+gp120) (in C).

Having established that WIN55,212-2 inhibits gp120-mediated suppression of DA uptake and blunts gp120-induced apoptosis, we then examined the involvement of specific cannabinoid receptors in mediating the neuroprotective effects of WIN55,212-2 by using the selective CB_1_ and CB_2_ receptor antagonists, SR141716A and SR144528, respectively. Our data show that the inhibition of gp120-mediated suppression of DA uptake (Figure 4A) and apoptosis (Figure 4B) by WIN55,212-2 involves CB_2_ receptors more than CB_1_ receptors. The involvement of CB_2_ receptors is further confirmed by the inhibition of gp120-induced apoptosis by the CB_2_ agonist JWH015, which is reversed in the presence of the CB_2_ receptor antagonists SR144528 (Figure 4C). 

**Figure 5:**
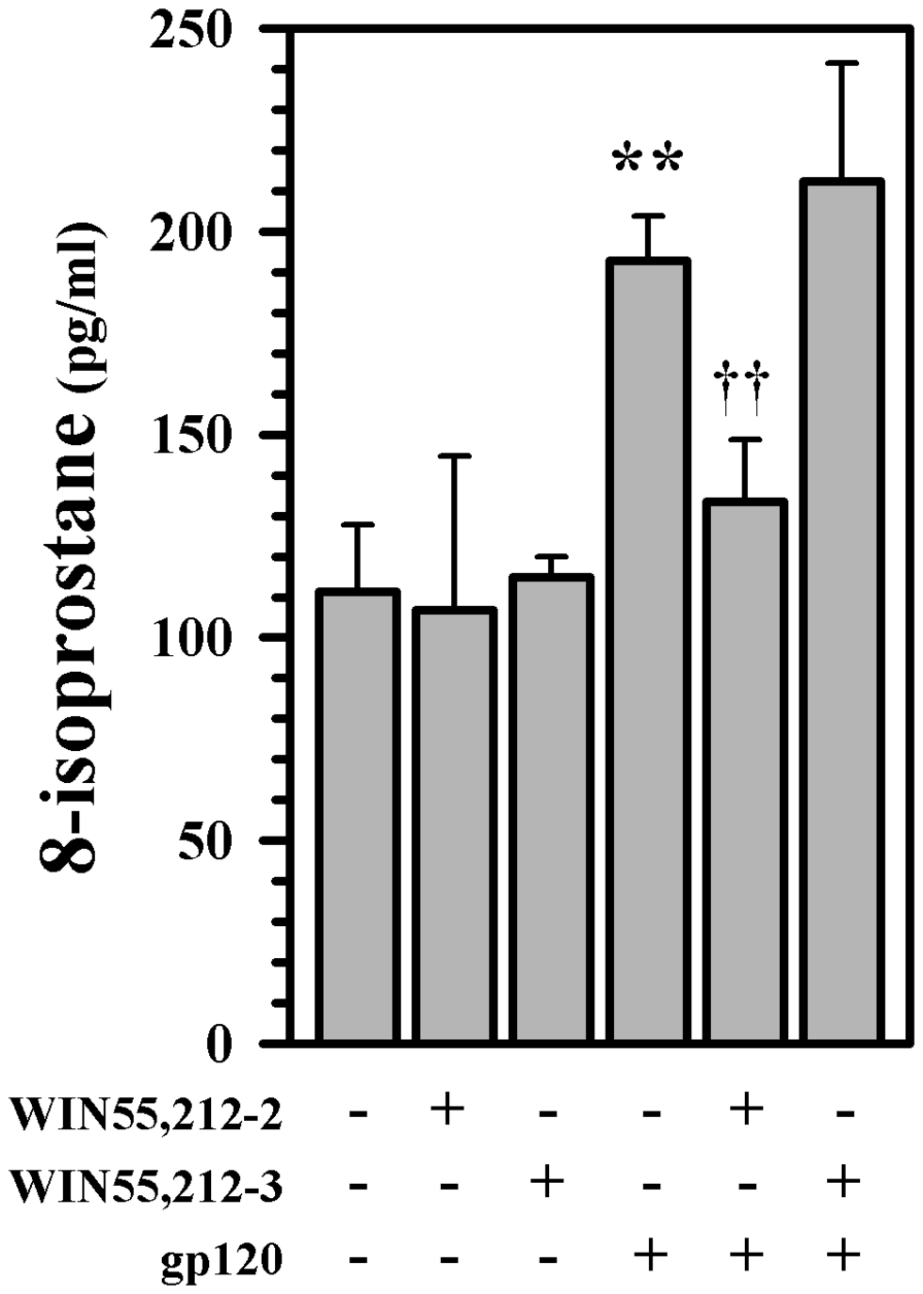
Blockade of gp120-induced lipid peroxidation. Human mesencephalic neuronal/glial cultures were untreated (C) or pretreated with WIN55,212-2 or WIN55,212-3 (0.3 μM) for 3h prior to gp120 (10 nM) treatment for 5 days followed by supernatant collection for 8-isoprostane assay. Data are mean ± SD of triplicates and representative of 3 separate experiments using different brain tissue specimens. **p<0.01 vs. control (C); ††p<0.01 vs. gp120.

**Figure 6:**
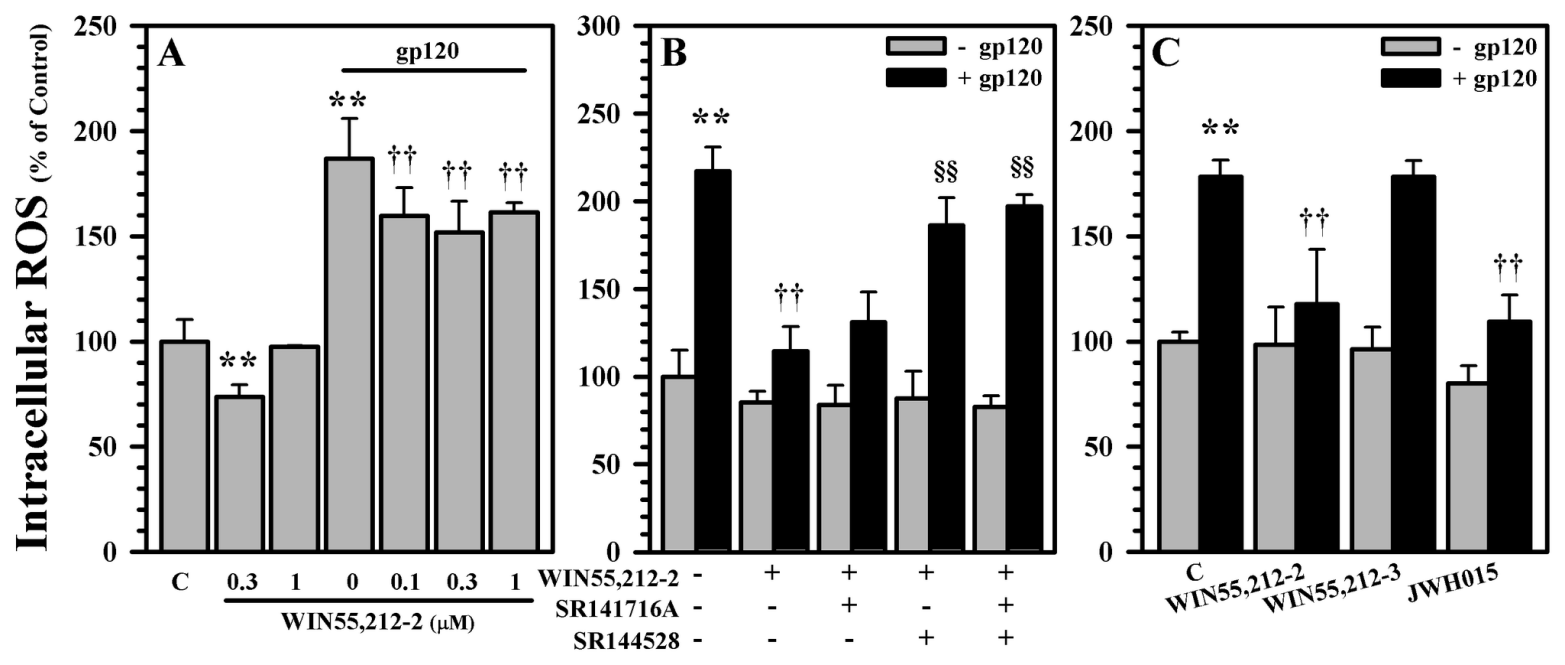
Reduction of gp120-induced intracellular ROS production. **A**) Human mesencephalic neuronal/glial cultures were untreated (C) or pretreated with WIN55,212-2 (0.1 to 1 μM) for 3h followed by gp120 (10 nM) treatment for 72h to measure production of intracellular ROS. **B**) Human mesencephalic neuronal/glial cultures were untreated (C) or pretreated with SR141716A (CB_1_ antagonist) or SR144528 (CB_2_ antagonist) (1 μM) for 30 min prior to WIN55,212-2 (0.3 μM) addition for 3h followed by gp120 (10 nM) treatment for 72h to measure production of intracellular ROS. **C**) Human mesencephalic neuronal/glial cultures were untreated (C) or pretreated with WIN55,212-2 (0.3 μM), WIN55,212-3 (0.3 μM), or JWH015 (CB2 agonist) (1 μM) for 3h prior to gp120 (10 nM) treatment for 72h to measure production of intracellular ROS. Data are mean ± SEM of triplicates of 3 separate experiments using different brain tissue specimens. **p<0.01 vs. control (C); ††p<0.01 vs. gp120; §§p<0.01 vs. WIN55,212-2+gp120.

Oxidative stress is considered a major contributor to HIV-1 neuropathogenesis. Previously, we showed that gp120 induces oxidative damage in our human mesencephalic neuronal/glial culture as demonstrated by enhanced levels of 8-isoprostanes (stable byproducts of oxidative damage to lipids), increased intracellular ROS, as well as evidence of the involvement of O_2_^-^ [41]. In support of the hypothesis that synthetic cannabinoids can reduce gp120-induced oxidative damage to dopaminergic neurons, we first quantified 8-isoprostane levels in our cultures. We found that exposure to gp120 resulted in increased 8-isoprostane levels (this effect was significant relative to heat-inactivated gp120 and anti-CXCR4 controls previously [41]) and that pretreatment of the cultures with WIN55,212-2 had a robust protective effect (Figure 5). WIN55,212-2 also abrogated gp120-induced production of intracellular ROS (Figure 6A), which through the use of CB receptor antagonists, appears to be primarily CB_2_ receptor mediated (Figure 6B). The same inhibitory effect is again confirmed by the inhibition of gp120-induced production of intracellular ROS by the CB_2_ agonist JWH015 (Figure 6C). 

**Figure 7:**
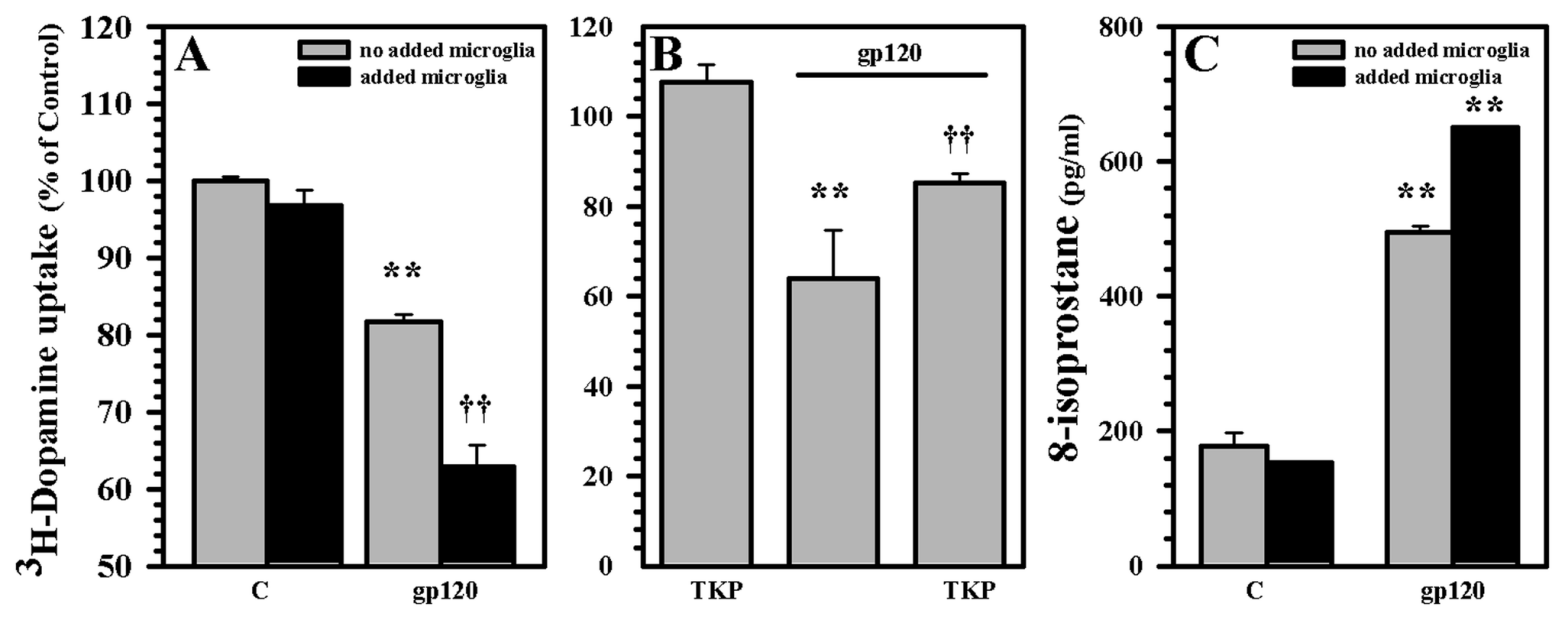
Potentiation of gp120-induced neuronal damage by human microglia. Additional human microglial cells (5x10^4^ cell/well) were added to human mesencephalic neuronal/glial cultures (5x10^5^ cell/well) for 3h followed by gp120 treatment for 5 d. DAT activity was measured by ^3^H-DA uptake assay **A**) with and without added microglia and **B**) in the presence of the tripeptide TKP, which inhibits microglial activation. **C**) Lipid peroxidation was measured by 8-isoprostane assay. Data are mean ± SD of triplicates and representative of 3 separate experiments using different brain tissue specimens. **p<0.01 vs. respective control; ††p<0.01 vs. gp120.

**Figure 8:**
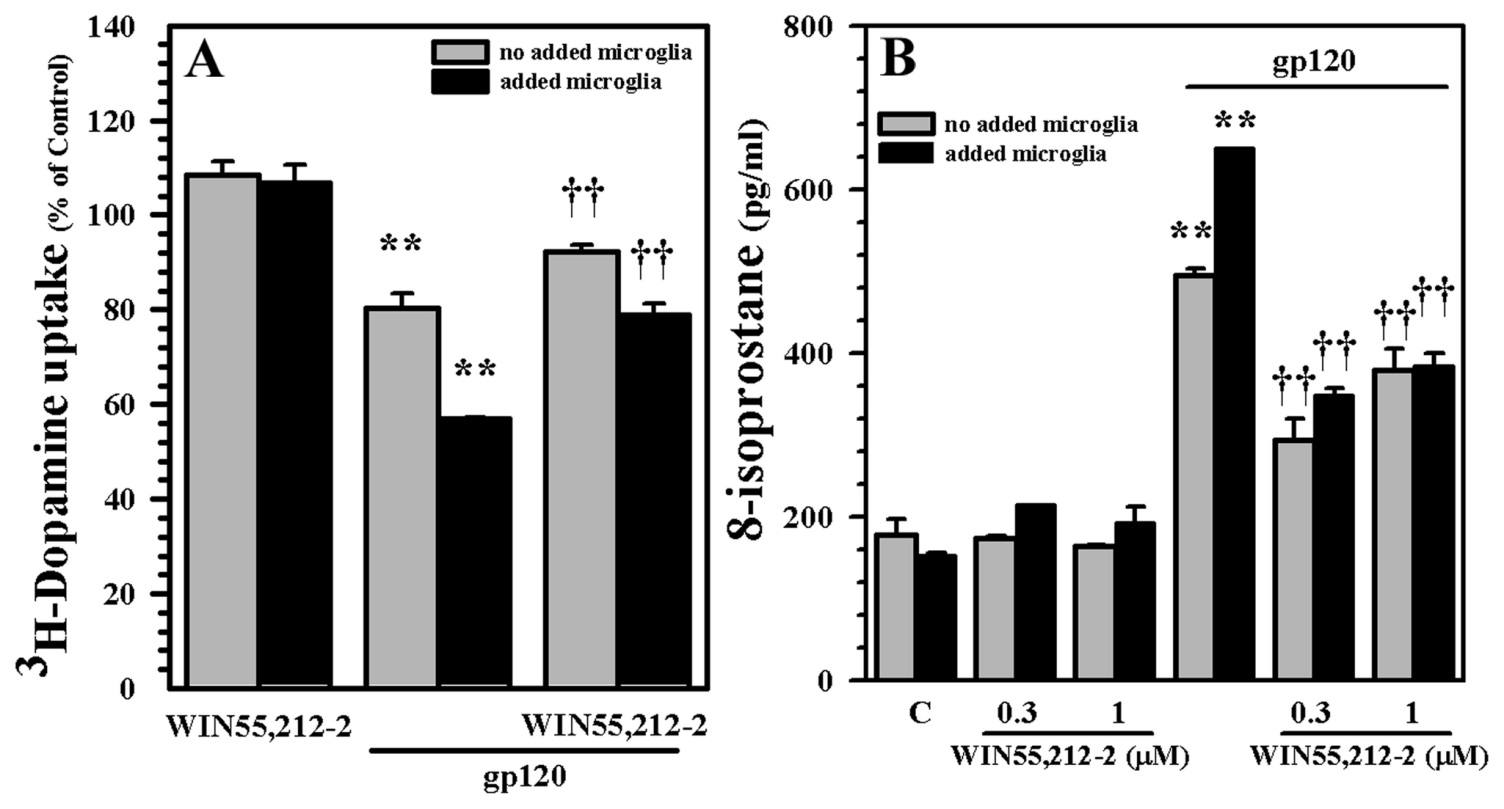
Blockade of microglial-enhanced gp120-induced decrease of DA uptake and increase of lipid peroxidation. Additional human microglial cells (5x10^4^ cells/well) were added to human mesencephalic neuronal/glial cultures for 3h followed by WIN55,212-2 (0.3 μM) pretreatment for 3h prior to gp120 treatment for 5 days. **A**) DAT activity was measured by ^3^H-DA uptake assay. **B**) Lipid peroxidation was measured by 8-isoprostane assay. Data are mean ± SEM of triplicates of 2 separate experiments using different brain tissue specimens. **p<0.01 vs. respective control, ††p<0.01 vs. gp120.

**Figure 9:**
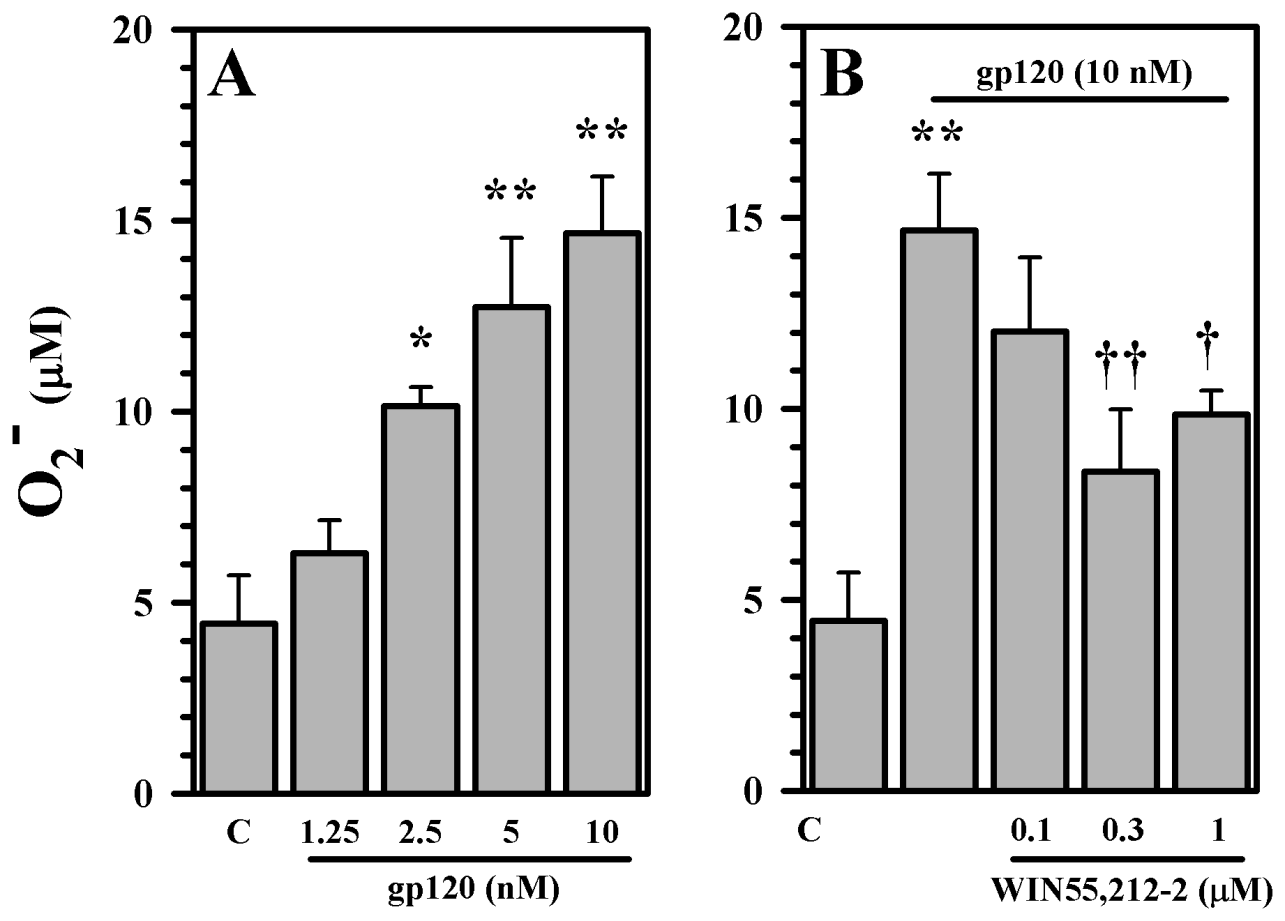
Blockade of gp120-induced superoxide production. Highly purified human microglial cell cultures were **A**) untreated (C) or treated with gp120 or **B**) untreated (C) or pretreated with WIN55,212-2 for 3h prior to gp120 treatment for 48h followed by measurement of O_2_^-^. Data are mean ± SD of triplicates and representative of 2 separate experiments using different brain tissue specimens. ^*^p<0.05, ^**^p<0.01 vs. control (C); ^†^p<0.05, ^††^p<0.01 vs. gp120.

Given that the principal source of the mediators of oxidative damage within the CNS are microglial cells, we next used primary cultures of highly purified microglial cells (>99% positive for the macrophage marker CD68) to investigate their potential role in gp120-indiced damage in our human mesencephalic neuronal/glial cultures. By adding supplementary purified human microglia to our cultures we established that human microglia markedly potentiate the neurotoxic effect of gp120 as measured by DA uptake (Figure 7A). In contrast, through the use of the tripeptide Thr-Lys-Pro (TKP), which inhibits microglial activation, the neurotoxic effect of gp120 in these cultures were diminished (Figure 7B). The addition of microglial cells to the model also increased oxidative damage, characterized by elevated 8-isoprostane levels (Figure 7C). We next performed similar experiments using additional purified human microglia in our cultures and examined whether WIN55,212-2 was capable of overcoming the enhanced gp120-induced neurotoxic effects. Pre-treatment with WIN55,212-2 was capable of blunting the enhanced damage caused by the addition of purified human microglia to our cultures exposed to gp120, as measured by DA uptake and 8-isoprostane levels (Figure 8A, B). Among the many ROS generated by activated microglia, we focused on O_2_^-^ for further evaluation. We found that gp120 stimulates production of O_2_^-^ in purified human microglial cells (Figure 9A) and that the generation of this ROS is inhibited by WIN55,212-2 (Figure 9B). 

**Figure 10:**
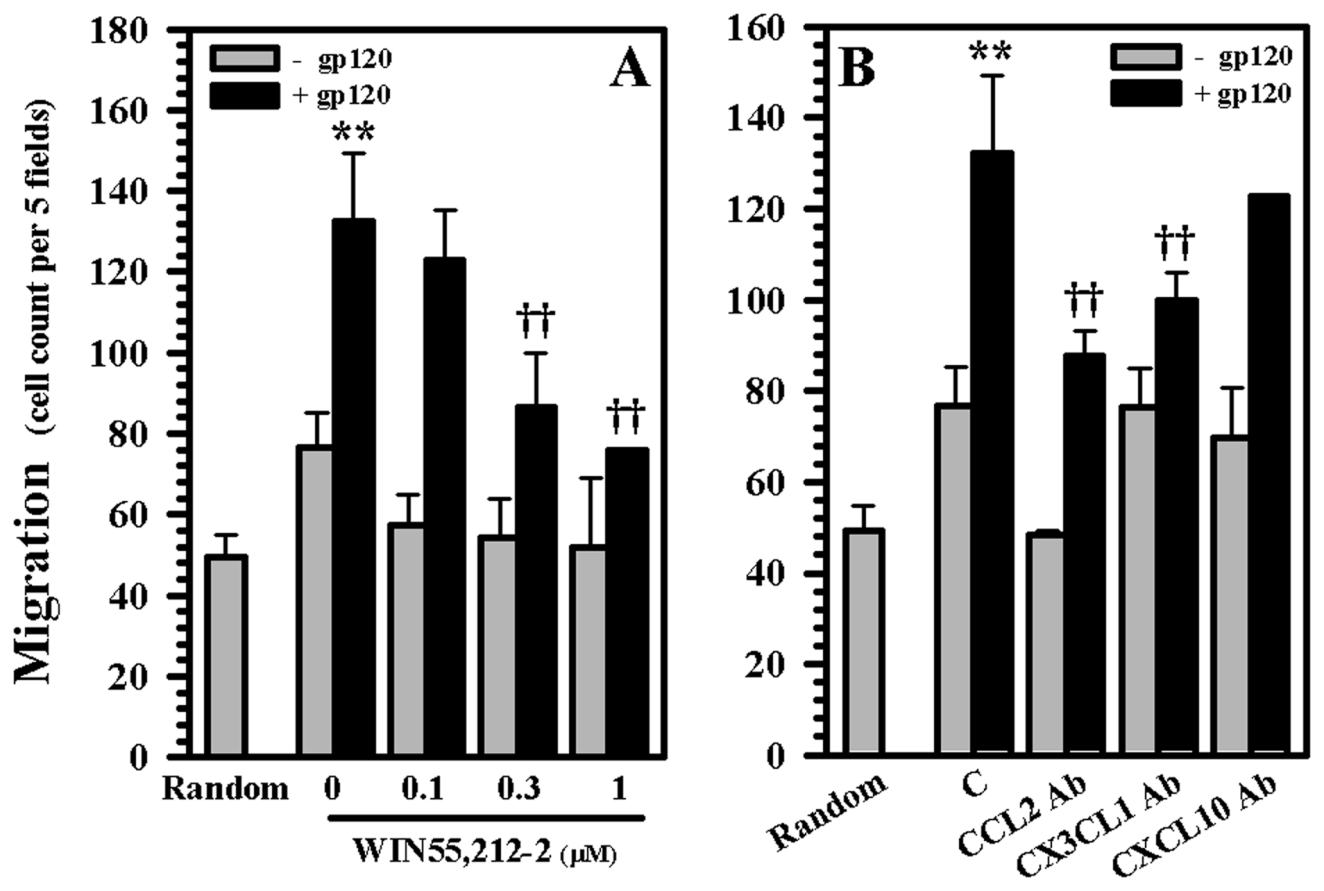
Blockade of human microglial migration towards supernatants from human mesencephalic neuronal/glial cultures. **A**) Human mesencephalic neuronal/glial cultures were pretreated with WIN55,212-2 (0.1 to 1 μM) for 3h followed by either no treatment or gp120 (10 nM) treatment for 72h. Supernatants were then collected for the chemotaxis assay. Highly purified human microglia were added to upper chambers with the lower chambers filled with supernatants from the human mesencephalic neuronal/glial cultures. After 3 h of incubation, human microglia that had migrated from upper chambers into lower chambers were quantified by Diff-Quik staining. **B**) Antibodies for chemokines CCL2, CX3CL1 and CXCL10 were added to collected supernatants for 30 min prior to chemotaxis assay. Data are mean ± SEM of triplicates of 3 separate experiments using different brain tissue specimens. **p<0.01 vs. control, ††p<0.01 vs. gp120.

**Figure 11:**
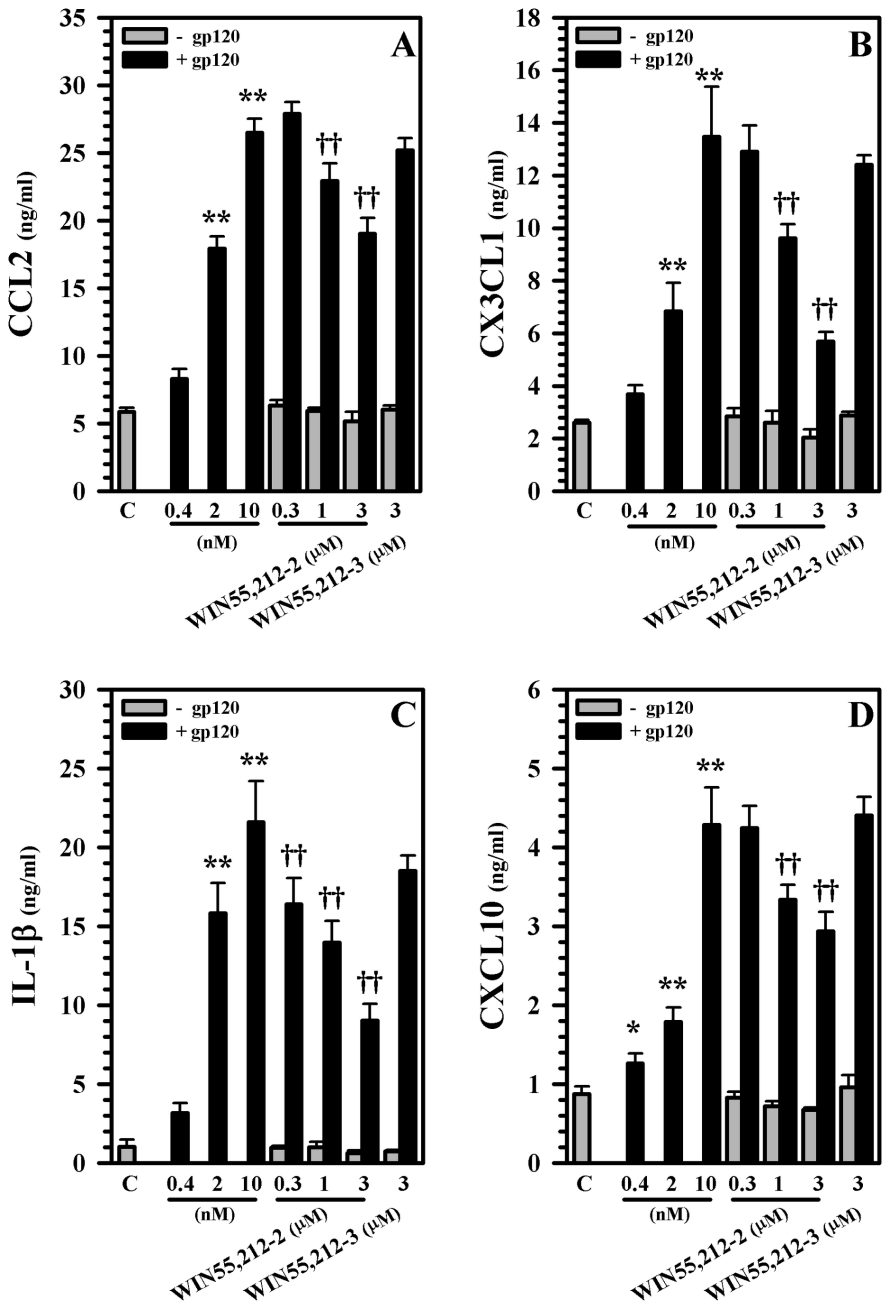
Blockade of gp120-induced production of CCL2, CX3CL1, IL-1β and CXCL10 in human mesencephalic neuronal/glial cultures. Human mesencephalic neuronal/glial cultures were pretreated with WIN55,212-2 (0.3 to 3 μM) or WIN55,212-3 (3 μM) for 3h prior to gp120 (10 nM) treatment for 72h followed by supernatant collection for measurement of **A**) CCL2, **B**) CX3CL1, **C**) IL-1β and **D**) CXCL10 production by ELISA. Data are mean ± SEM of triplicates of 2 separate experiments using different brain tissue specimens. *p<0.05, **p<0.01vs. control (C); ^††^p<0.01 vs. gp120.

**Figure 12:**
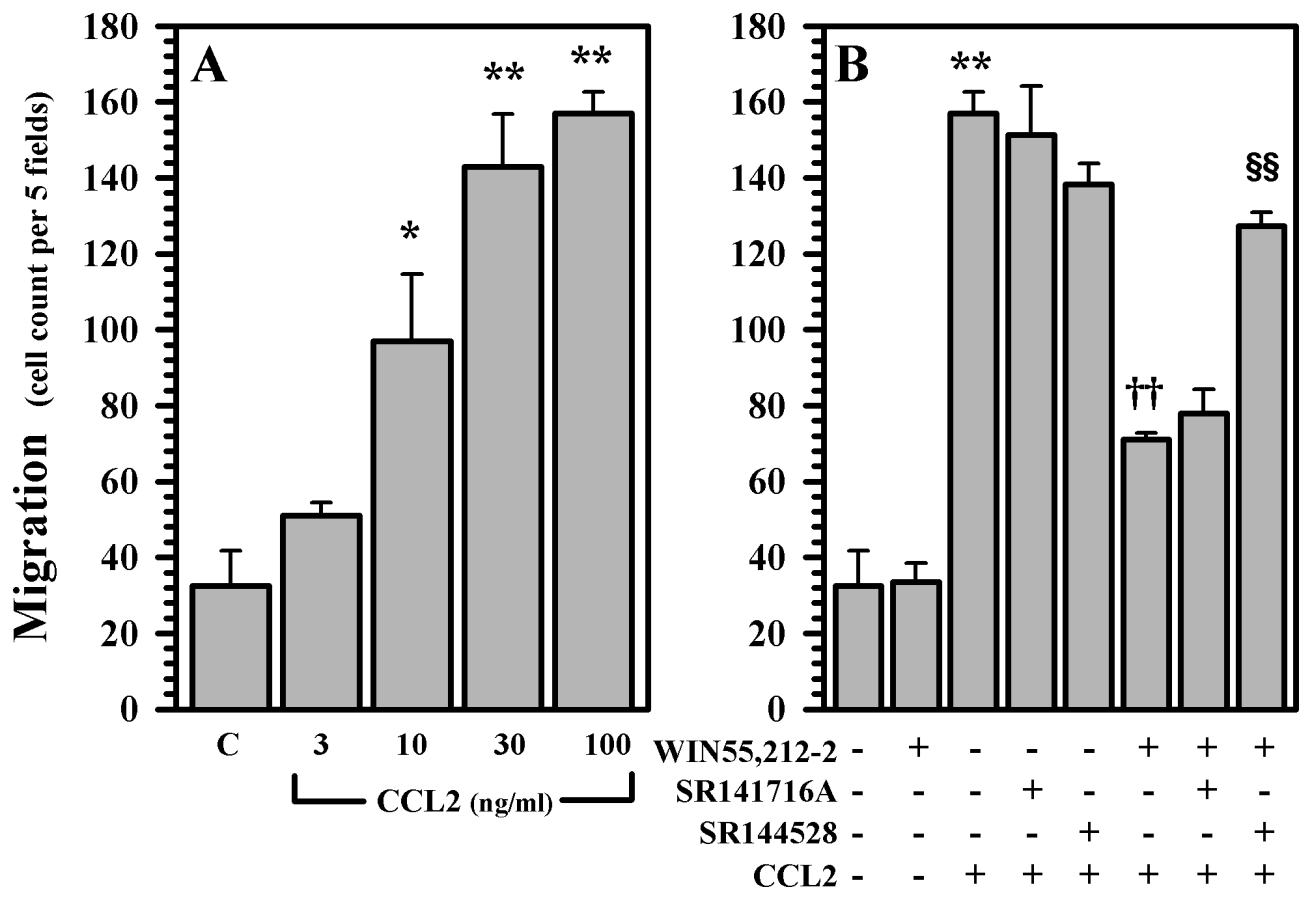
CB_2_ receptor-mediated microglial migration. A) Human microglia were loaded onto the upper chemotaxis chambers with lower chambers filled with media (C) or CCL2 (3 to 100 ng/ml) and incubated for 3h. B) Human microglia were loaded onto upper chemotaxis chambers and pretreated with CB antagonists (SR141716A or SR144528, 3 µM) for 30 min followed by WIN55,212-2 (1 µM) treatment for 3h prior to be assembled with lower chambers filled with media (C) or CCL2 (100 ng/ml) and incubated for 3h. Migrated microglial cells were quantified by Diff-Quik staining. *p<0.05, **p<0.01 vs. control (C); ^††^p<0.01 vs. CCL2; ^§§^ p<0.01 vs. WIN55,212-2+CCL2.

Since the presence of additional human microglia markedly potentiates the neurotoxic effect of gp120 in our human mesencephalic neuronal/glial cultures, we next examined the capabilities of WIN55,212-2 in blocking the recruitment of additional microglia to our cultures in an effort to model the effects of WIN55,212-2 on microglial recruitment to the human mesencephalon. WIN55,212-2 was able to inhibit the migration of highly purified human microglia towards the supernatants generated from our gp120-exposed human mesencephalic neuronal/glial cultures in a dose dependent fashion (Figure 10A). Among the three specific chemokines that we analyzed (CCL2, CX3CL1, and CXCL10), CCL2 and CX3CL1 appeared to be the most important chemokines in attracting human microglia generated by our cultures after exposure to gp120 (Figure 10B). Next we examined the impact of WIN55,212-2 on the generation of chemokines CCL2, CX3CL1, and CXCL10 and cytokine interleukin (IL)-1β in our cultures after exposure to gp120. WIN55,212-2 was able to inhibit the production of each of these chemokines/cytokine in a dose dependent fashion (Figure 11). Finally, we singled out CCL2 and evaluated the effect of WIN55,212-2 on the migration of microglial cells towards this chemokine. CCL2 was effective in attracting highly purified human microglia in a dose-dependent manner (Figure 12A). WIN55,212-2 was able to inhibit the migration of microglia towards CCL2; this process was reversed in the presence of the CB_2_ receptor antagonists SR144528, suggesting that this inhibition is principally CB_2_ receptor mediated (Figure 12B). 

## Discussion

In this study, using a human *in vitro* model of the mesencephalic region of the brain, we show that in the presence of the synthetic cannabinoid WIN55,212-2, the damaging effects of the HIV-1 viral protein gp120 on this region, and on dopaminergic neurons specifically, are ameliorated. WIN55,212-2 was able to ameliorate the diverse effects of gp120 on dopamine uptake, apoptosis, and oxidative damage in our model. The identification that CB_2_ receptors were the principal mechanism by which WIN55,212-2 exerted its effect implies a significant role for microglial cells. The addition of purified human microglial cells to these cultures markedly potentiated gp120-induced adverse effects; WIN55,212-2 blocked these toxic effects of gp120, which supports the hypothesis that the neuroprotective effects of synthetic cannabinoids are produced, at least in part, through mechanisms involving microglial cells. In studies of purified microglia, gp120 stimulated O_2_^-^ release, which we speculate contributes to oxidative damage of dopaminergic neurons; WIN55,212-2 was found to inhibit gp120-induced O_2_^-^ release by microglia. WIN55,212-2 was also capable of inhibiting the migration of human microglia towards the supernatants generated from our gp120-exposed cultures, inhibited the production of CCL2, CX3CL1, CXCL10 and IL-1β generated from our cultures, and inhibited the migration of human microglia towards CCL2. Overall, these series of experiments support the hypothesis that synthetic cannabinoids are capable of protecting human dopaminergic neurons from gp120, principally through mechanisms involving CB_2_ receptors and microglia.

Of the two classical cannabinoid receptors, CB_1_ receptors are expressed primarily in the brain, and are abundantly expressed in the basal ganglia and hypothalamus. Activation of these receptors by natural cannabinoids, such as Δ^9^-tetrahydrocannabinol (THC), as well as by synthetic cannabinoids has been extensively investigated [59]. CB_2_ receptors are primarily expressed in cells of the immune system, including microglia [60,61]. The specific protective effects of cannabinoids on neuronal cell damage have been extensively studied in rodent models [60,62-65]. These studies show that both CB_1_ receptor-mediated [66-68] and non-receptor-mediated i.e. via an antioxidant effect of cannabinoids [69-71] mechanisms are involved in the neuroprotective properties of cannabinoids. Despite this evidence from rodent models, little is known about the neuroprotective effects of cannabinoids on human neurons, especially as these effects may pertain specifically to neuroprotection against HIV-1. 

We attempted to explore the neuroprotective role of cannabinoids in a model of HIV neuropathogenesis by investigating several aspects of gp120-induced damage to dopaminergic neurons; these included numerical loss, morphological changes, loss of DA transporter function, neuronal apoptosis, and oxidative damage. As we previously reported, structural damage to dopaminergic neurons was noted in response to gp120 in both loss of numbers, loss of dendrites, and decreased DA transport function [41]. In support of the hypothesis that synthetic cannabinoids have a neuroprotective influence, treatment of these neuronal cultures with the CB_1_/CB_2_ receptor ligand WIN55,212-2 preserved the numbers of neurons and the morphologic structure of the dendrites. Similar findings of enhanced survival and preservation of TH^+^ neuronal morphology by WIN55,212-2 has also been recently demonstrated in the substantia nigra of rat brain stimulated with lipopolysaccharide (LPS) [72]. However, Δ^9^-tetrahydrocannabinolic acid (THCA) and Δ^9^-tetrahydrocannabinol (THC) were unable to preserve neurite outgrowth in dopaminergic neurons in a murine 1-methyl-4-phenylpyridinium (MPP^+^) model [73]. Other investigators, using significantly smaller concentrations of gp120, have shown that gp120 induces significant synaptic loss in rat hippocampus, while having no effect on neuronal survival. It is postulated that synaptic loss precedes cell death and that different pathways lead to either synaptic loss or cell death, which may explain the lack of cell death in this model in which lower concentrations of gp120 were used. Regardless, WIN55,212-2 inhibited gp120-induced synaptic loss in this model as well [74]. 

Because DA transporters are significantly reduced in HAND patients compared with seronegative controls [38] we examined the effect of WIN55,212-2 on gp120-induced inhibition of DA transporter function. We found that gp120 decreased DA uptake and that treatment with WIN55,212-2 significantly reduced (by 39.07 % ± 5.07%) this toxic effect of gp120. This protective effect of WIN55,212-2 was dose dependent and involved the CB_2_ receptor. The impairment of ^3^H-DA uptake by dopaminergic neurons in our study could be the result of either an alteration of K_m_ or V_max_. Although definitive studies to resolve this were not performed, a blunting of dopaminergic neurites was observed in similar fashion to that seen in a rat model [72], suggesting that the impaired ^3^H-DA uptake was due to an altered V_max_. Finding that WIN55,212-2 preserves the number of human dopaminergic neurons, the morphologic structure of the dendrites, and attenuates the loss of DA transporter function in our model, demonstrates that the neuroprotective properties of cannabinoids established in animal models extends to human cells stimulated by the important HIV-1 viral protein gp120.

Neuronal apoptosis is one of the histopathological hallmarks of HAD and other investigators have reported apoptosis of rodent dopaminergic neurons after gp120 exposure, which appears to be caspase-3-related [75]. We have evaluated the gp120-induced apoptosis in our model previously [41]; the neuroprotective effect of WIN55,212-2 on gp120-induced apoptosis was assessed in this study. We exposed our human mesencephalic neuronal/glial culture to gp120 and assessed the level of neuronal apoptosis, both as non-specific apoptosis and apoptosis of dopaminergic neurons. Supporting our hypothesis, gp120-induced apoptosis of dopaminergic neurons was significantly reduced by WIN55,212-2. Experiments involving WIN55,212-3 (inactive enantiomer), JWH015 (CB_2_ agonist) and SR144528 (CB_2_ antagonist) validated that the effect of WIN55,212-2 principally involves CB_2_ receptors.

Oxidative stress is considered a major contributor to HIV-1 neuropathogenesis [76-83]. Previously, in support of the hypothesis that gp120 induces oxidative damage to dopaminergic neurons, we demonstrated enhanced levels of 8-isoprostanes (stable byproducts of oxidative damage to lipids), increased intracellular ROS, as well as evidence of the involvement of O_2_^-^ [41]. While many oxidative products may be involved in oxidative damage in our mesencephalic neuronal/glial cultures, it is likely that free radicals generated downstream from O_2_^-^ have important deleterious effects on dopaminergic neurons. In our current study, pretreatment of the cultures with WIN55,212-2 had a robust protective effect against gp120-induced oxidative damage as measured by lipid peroxidation and intracellular ROS. The source of oxidative stress within our culture model could be astrocytes and/or microglia. Experiments involving WIN55,212-3 (inactive enantiomer), JWH015 (CB_2_ agonist) and SR144528 (CB_2_ antagonist) validated that the effect of WIN55,212-2 on oxidative stress by these measures in our cultures principally involves CB_2_ receptors. Therefore, it is suggested that the neuroprotective effect of cannabinoids against oxidative damage is operating principally through microglia rather than astrocytes or neurons directly, since latter two do not possess CB_2_ receptors [84]. 

While astrocytes play an important role in oxidative damage within the CNS [85,86], microglial cells are one of the principal cell types involved in neuroinflammation and oxidative stress within the CNS. For example, microglia are the most capable brain cell type for generating large quantities of the free radical O_2_^-^ [8]. Murine models of Parkinson’s disease have demonstrated that selective toxicity of dopaminergic neurons to LPS or MPP^+^ involve microglial-derived O_2_^-^ [87,88]. Microglia-derived ROS mediate the loss of nigral dopaminergic neurons *in vivo* and *in vitro* [88-90]. Microglia also contribute to the damage of dopaminergic neurons in SIV models as well [39,40]. Non-selective cannabinoid agonists WIN55,212-2 and HU-210 increase survival of dopaminergic neurons in the substantia nigra of the rat exposed to LPS via inhibition of microglial NADPH oxidase, ROS production, and proinflammatory cytokines tumor necrosis factor (TNF)-α and IL-1β [72]; WIN55,212-2 also inhibits MPTP-derived dopaminergic neuronal loss in murine substantia nigra and ventral midbrain via CB_2_-mediated inhibition of microglial activation [46]. Finally, cannabidiol inhibited microglia activation *in vitro* and improved learning behavior in an *in vivo* model of AD [91]. Combined, these results support the prospect that beneficial effects of cannabinoids may come from their ability to suppress microglial activation. 

One noteworthy feature of the midbrain and the substantia nigra specifically that may help explain the susceptibility of this brain region to HIV-1-related damage is that this area contains a high concentration of microglial cells [92], which are the only brain cell types that can support productive HIV-1 infection, express CXCR4 and CCR5 and are a rich source of inflammatory mediators (O_2_^-^ and cytokines/chemokines) [8-11]. Previously, we have shown that primary cultures of highly purified human microglial cells (>99% positive for the macrophage marker CD68) also express CB_1_ and CB_2_ receptors and that the CB_1_/CB_2_ receptor agonists WIN55,212-2 and CP55,940 as well as the CB_2_ receptor selective agonist JWH015, inhibit expression of HIV-1 in these cells, suggesting that this antiviral property involves CB_2_ receptor activation [48,49]. 

In this study, we demonstrated that the addition of purified human microglia to our human mesencephalic neuronal/glial culture exacerbates gp120-induced inhibition of DA uptake in dopaminergic neurons, suggesting that additional sources of inflammation and oxidative stress in the setting of gp120 stimulation enhance downstream neuronal damage. Others have shown that the tripeptide TKP, which inhibits microglial activation [22,93-96], reduces gp120-induced synaptic loss in rat hippocampus cultures [74]. By inhibiting microglial activation with TKP in our model, dopaminergic neuronal function as measured by DA uptake, improves. Similarly, WIN55,212-2 partially reversed gp120-induced inhibition of DA uptake in dopaminergic neurons; an effect that is more robust in the presence of additional purified human microglial cells. Given the notion that activated microglia are a likely source of ROS, we also demonstrated that the addition of purified human microglia markedly increased the level of lipid peroxidation as measured by 8-isoprostane in the setting of gp120 exposure. Among the many ROS generated by activated microglia, we focused on O_2_^-^ for further study. We found that gp120 stimulates production of O_2_^-^ in purified human microglial cells and that the generation of this ROS is inhibited by WIN55,212-2. The results from these purified human microglia experiments function as a proof of principle that synthetic cannabinoids can directly inhibit an important microglia-derived ROS species, which supports the hypothesis that synthetic cannabinoids are neuroprotective due to their effects on microglial cells. 

Since the presence of additional human microglia markedly potentiates the neurotoxic effect of gp120 in our human mesencephalic neuronal/glial cultures, we next examined the capabilities of WIN55,212-2 in blocking the recruitment of additional microglia to our cultures in an effort to model the effects of WIN55,212-2 on microglial recruitment to the human mesencephalon. WIN55,212-2 was able to inhibit the migration of highly purified human microglia towards the supernatants generated from our gp120-exposed human mesencephalic neuronal/glial cultures in a dose dependent fashion. Among the many chemokines we could have examined, we focused on three specific chemokines (CCL2, CX3CL1, and CXCL10); of these, CCL2 and CX3CL1 appeared to be the most effective chemokines in attracting human microglia. CCL2, which is up-regulated in activated microglia and constitutively produced by neurons [97], plays a central role in HAND by attracting microglia [98] and monocytes [99-101] into the central nervous system and is considered a marker of poor prognosis [102-105]. CX3CL1 and its receptor CX3CR1 have received increased attention regarding their roles in HIV-1 infection [106-108] and in HAND specifically [106,109,110]. CX3CL1 is expressed by neurons and astrocytes [111], where it induces microglial proliferation [111] and migration [112]. Inflammatory factors such as TNF-α and interferon (IFN)-γ synergistically enhance CX3CL1 expression by human astrocytes [113]. Functionally, CX3CR1 is constitutively expressed in microglia and neurons [111]. The finding of elevated cerebrospinal fluid levels of CX3CL1 in HAND suggests that it may play a role in HIV neuropathogenesis [114,115]. CXCL10 has chemoattraction for monocytes/macrophages/microglia, is upregulated in human astrocytes exposed to HIV viral protein Tat, IFN-γ and TNF-α [116], is elevated in the cerebrospinal fluid levels of HAND patients and contributes to HIV-1 neurotoxicity [117]. WIN55,212-2 was able to inhibit the production of chemokines CCL2, CX3CL1, and CXCL10 and cytokine IL-1β in our cultures after exposure to gp120 in a dose dependent fashion. WIN55,212-2 was also able to inhibit the migration of highly purified human microglia towards CCL2 via the CB_2_ receptor. By limiting the migration of additional activated microglia and/or limiting the stimulation of surrounding astrocytes by IL-1β, these data suggests an additional mechanism by which synthetic cannabinoids reduce the exposure of dopaminergic neurons to further neurotoxic insult. Others have described similar findings in non-human models. In a neonatal rat middle cerebral artery occlusion (MCAO) model, mRNA expression of CB_2_ receptor (but not CB_1_ receptor), chemokine receptors (CCR2 and CX3CR1), cytokines (IL-1β and TNF-α), as well as protein expression of chemokines CCL1 and CCL3 and microglial activation all increased after MCAO. WIN55,212-2 reduced microglial activation, attenuated infarct volume, and microglial accumulation in the injured cortex after MCAO [118]. Cannabinoids also inhibit migration of murine BV-2 microglial-like cells to the HIV protein Tat via CB_2_ [119]. Our findings also agree with other studies showing that cannabinoid agonists can modulate microglia migration via activation of CB_2_ receptor *in vitro* [65] and *in vivo* [91]. Thus, WIN55,212-2 may exert its neuroprotective actions by reducing the deleterious effects triggered by microglial activation/infiltration, which ultimately contribute to the demise of dopaminergic neurons.

For all of our experiments, we focused on the effects of synthetic cannabinoids on gp120_LAV(IIIB)_. The restriction of our experiments to gp120 from a single, CXCR4-tropic variant when different HIV-1 gp120 variants may occur in the brain limits the generalizability of our findings. While the effects of synthetic cannabinoids were demonstrated for gp120_LAV(IIIB)_, this may not hold true for CCR5-tropic variants. The rationale for selecting gp120_LAV(IIIB)_ includes maintaining consistency with our previous work which identified the preferential sensitivity of DA neurons to gp120-induced toxicity [41] and the fact that virtually every investigator looking into gp120-induced toxicity in the brain uses T-trophic strains gp120_SF2_ or gp120_IIIB_ [21-23,56-58,75,83,120,121]. This also includes the gp120 transgenic mouse model [122]. CCR5-tropic viruses predominate in the initial stage of infection, whereas a switch from CCR5- to CXCR4-tropic viruses occurs in the late stages of infection in a subset of patients. In patients, the emergence of CXCR4-tropic virus usually occurs after years of infection and correlates with more rapid progression to AIDS. Consensus suggests that T-tropic strains that appear late in the course of infection might cause neuronal apoptosis and dementia. The late-stage patients who frequently harbor CXCR4-tropic virus are also the most likely to benefit from cannabinoid treatment. 

Another limitation to our study centers on the uncertainty of the local concentration gp120 in the human mesencephalon. Although it is difficult to determine the concentration of gp120 *in vivo*, we used a concentration that is in the range used by several other investigators [14,56-58]. HIV gp120 has been detected at levels between 200 pg/mL and 2000 pg/mL in the peripheral blood. HIV-1 gp120 accumulated in the lymph nodes of infected animals and that a steep concentration gradient existed between the high concentrations (218 ng/mL) detected in lymph node lysates and the significantly lower concentrations (1.5 ng/mL) detected in the serum of animals with high viral loads [123]. We do not feel that serum levels of gp120 are a good indicator of its concentration in tissues. Much like the example of heightened concentration of HIV-1 gp120 in lymphoid tissue, the level of gp120 is likely to be much more concentrated around cells that are productively infected, such as microglia, leading to high local concentrations that may be intensified by binding to extracellular matrix components. Since our experiments show that the effects of synthetic cannabinoids are primarily working through CB_2_ receptors on microglial cells, they would be operating in this local environment. However, because of the obvious uncertainty of the true concentration of gp120 in the local environment of the human mesencephalon, our data must be evaluated in the context of this ambiguity. 

Overall, this study confirms that gp120 is capable of damaging human dopaminergic neurons, that this damage involves human microglia, and that synthetic cannabinoids, such as WIN55,212-2, can alleviate this damage through mechanisms involving human microglia. Thus, the results of these experiments set the stage for further studies designed to tease out the role human microglia have in the mechanisms underlying the toxic effects of HIV-1 on human dopaminergic neurons and understanding the microglial-centered mechanisms underlying the protective effects of selected synthetic cannabinoids.

## Materials and Methods

### Reagents

The following reagents were purchased from the indicated sources: Recombinant HIV-1 gp120_LAV_ (IIIB), which has clade B origins (Protein Sciences, Meriden, CT); cannabinoid agonists and antagonists: WIN55,212-2, WIN55,212-3 (S(-)-[2,3-dihydro-5-methyl-3-[(4-morpholinyl)methyl]pyrrolo-[1,2,3-de]-1,4-benzoxazinyl]-(1-naphthalenyl) methanone mesylate, an inactive enantiomer of WIN55,212-2), CP55,940 ((-)-*cis*-3-[2-hydroxy-4-(1,1-dimethylheptyl)phenyl]-trans-4-(3-hydroxypropyl) cyclohexanol) and JWH 015 ((2-methyl-1-propyl-1*H*-indol-3-yl)-1-naphthalenylmethanone) (Tocris, Ellisville, MO), SR141716A (5-(4-chlorophenyl)-1-(2,4-dichlorophenyl)-4- methyl-*N*-(1-piperidyl)pyrazole-3-carboxamide hydrochloride), SR144528 ((1*S*-endo)-5-(4-chloro-3-methylphenyl)-1-((4-methylphenyl)methyl)-*N*-(1,3,3-trimethylbicyclo(2.2.1)hept-2-yl)-1*H*-pyrazole-3-carboxamide) (NIDA, National Institutes of Health, Bethesda, MD); Dulbecco’s modified Eagle’s medium (DMEM), Hank’s balanced salt solution (HBSS), penicillin, streptomycin, trypsin, glucose, bovine serum albumin, polyoxyethylenesorbitan monolaurate (Tween 20), PBS, Krebs-Ringer buffer, paraformaldehyde, uridine, fluorodeoxyuridine, poly-D-lysine, superoxide dismutase (SOD) and mazindol (Sigma-Aldrich, St. Louis, MO); rabbit anti-tyrosine hydroxylase (TH) antibody (Pel-Freez, Rogers, AR); fetal bovine serum (FBS) (Hyclone Laboratories, Logan, UT); rabbit anti-dopamine β-hydroxylase (DBH) antibodies (Millipore, Billerica, MA); mouse anti-CD68 antibody (DAKO, Carpinteria, CA); donkey anti-rabbit or anti-mouse IgG rhodamine- conjugated secondary antibodies (Jackson ImmunoResearch, West Grove, PA); propidium iodide (PI) and Hoechst stain 33342 (Roche, Indianapolis, IN); [7,8-^3^H]dopamine (DA, 50 Ci/mmol) (GE Healthcare, Piscataway, NJ). 

### Assay kits

The following kits were purchased from the indicated sources: ApopTag^®^ in situ apoptosis detection kit (formerly Chemicon, now Millipore); cell death detection kit (Roche); 8-isoprostane EIA kit (Cayman Chemical, Ann Arbor, MI); Vectastain^®^ ABC kit (Vector Laboratories, Burlingame, CA).

### Mesencephalic neuronal/glial culture

Human fetal brain tissues were obtained from women undergoing elective abortion, in accordance with informed-consent guidelines and a protocol approved by the Human Subjects Research Committee at the University of Minnesota. The donors have first signed a consent form for the elective abortion and then are asked if they would donate the tissue for medical research; if the donors wish to donate the tissue, then a separate consent form, approved by the University of Minnesota Human Subjects Research Committee, is signed. No additional procedures or inducements are used to obtain these tissues, that ordinarily would be discarded, and no records are kept by our research group that would reveal the donor’s identity or demographic information. Mesencephalic neuronal/glial cultures were prepared from 8- to 12-week-old aborted human fetal brain tissues as described previously [52,53]. In brief, ventral mesencephalon tissues were dissected and incubated in 0.1% trypsin for 15 min at 37°C followed by washing twice with DMEM containing 10% FBS. After gentle mechanical trituration with pasture pipette, single cell suspensions in DMEM containing 10% FBS and penicillin/streptomycin (100 U/ml/100 μg/ml) were stained with trypan blue to confirm cell viability before being plated onto poly-D-lysine-coated culture plates (5x10^5^ cells/well or 1x10^5^ cells/well in 24 or 96-well plates, respectively) or 4-well chamber slides (4x10^5^ cells/well). On day 5, cell cultures were treated with uridine (33.6 μg/ml) and fluorodeoxyuridine (13.6 μg/ml) followed by media replacement with DMEM and 10% FBS on day 6 and every 4 d thereafter. Presence of dopaminergic neurons was verified by ^3^H-DA uptake and immunocytochemical staining of tyrosine hydroxylase (TH). Twelve-day-old mesencephalic neuronal/glial cultures were used for all experiments. Because the nigrostriatal pathway expresses CXCR4, we elected to use the CXCR4-specific gp120_LAV_ (IIIB) for all experiments [124].

### Microglial cell preparations

Primary human microglial cell cultures were prepared as previously described [125,126]. Human brain tissues were obtained under a protocol approved by the Human Subjects Research Committee at our institution. Briefly, brain tissues from 16- to 22-week-old fetuses were dissociated by trypsinization (0.25%) for 30 min and plated into 75-cm^2^ tissue culture flasks in DMEM containing 10% FBS, penicillin (100 U/mL), and streptomycin (100 µg/mL). Cells were incubated for 10-14 days with weekly medium changes. Microglial cells floating in the medium of mixed cultures were collected, centrifuged, and reseeded in 96-, 24-, 12-, and 6-well tissue culture plates (1x10^4^, 2x10^5^, 1x10^6^, and 2.5x10^6^ cells/well, respectively) with fresh medium. The cultures were washed 1h after seeding to remove non-adherent cells. Purified microglia are composed of a cell population of which >99% stain with anti-CD68 antibody (a human macrophage marker) and <1% stain with anti-GFAP antibody (an astrocyte marker).

### Immunocytochemical staining

For neuronal staining, monoclonal anti-NeuN or anti-MAP-2 antibodies were used. To identify dopaminergic neurons, polyclonal anti-TH antibody were used. Microglia were detected with anti-CD68 antibody. Following previously described procedures [56,127], mesencephalic neuronal/glial cell cultures grown in 24-well plates or 4-well chamber slides were fixed with 4% paraformaldehyde for 20 min followed by washing with PBS and incubation with 10% normal goat or donkey serum containing 0.3% triton X 100 in PBS for 1h at room temperature (RT). After washing, primary antibodies were added and incubated overnight at 4°C (anti-TH 1:1000, and anti-CD68 1:100). After washing 4 times, secondary antibodies were added (donkey anti-mouse or rabbit rhodamine-conjugate) for 1h at RT. To visualize gp120-induced morphology damage, cell cultures stained with anti-TH antibody were incubated with biotinylated goat anti-rabbit IgG for 60 min followed by addition of Vectastain ABC reagents and color development with 3,3’-diaminobenzidine (DAB) (Vector Laboratories). 

### Dopamine transporter activity assay

A high affinity transporter activity assay was performed as previously described [56,128]. After treatment, mesencephalic neuronal/glial cell cultures were washed 3 times with DMEM or Krebs-Ringer buffer. For DA uptake, cultures were incubated with ^3^H-dopamine (50 nM) in DMEM for 10 min at 37°C. After washing 3 times with cold DMEM, cells were lysed in 2N NaOH (300 µl) followed by lysate collection into scintillation cocktail for ^3^H radioactivity counting. Non-specific dopamine uptake was observed in the presence of mazindol (10 μM).

### Assessment of lipid peroxidation

All reactive oxygen species themselves are short lived due to their highly reactive nature. For this reason, oxidative tissue damage is generally analyzed through measurement of secondary products indicative of oxidative damage. We assessed the level of oxidative damage to lipids using an 8-isoprostane EIA kit to quantify the level of lipid peroxidation in samples and performed according to the manufacturer’s instructions (Cayman).

### Cell death detection ELISA

Quantitative *in vitro* determination of cytoplasmic histone-associated DNA fragments after induced cell death was performed according to manufacturer’s protocol. To measure apoptosis, cell lysates from untreated control or treated cell cultures in 24-well plates were added to the streptavidin-coated 96-well ELISA plates together with anti-histone-biotinylated and anti-DNA-peroxidase antibodies. After incubation and washing, DNA fragments were captured and detected by a chromogenic enzyme-substrate reaction [129].

### Intracellular ROS assay

The production of intracellular ROS in human mesencephalic neuronal/glial cultures treated with gp120 was measured by 2’,7’-dichlorodihydrofluorescin diacetate (H_2_DCFDA; formerly Calbiochem, now EMD, Gibbstown, NJ) as previously described [87]. Human mesencephalic neuronal/glial cultures (1x10^5^ cells/well) were incubated with gp120 (0.4 to 10 nM) for 5 days followed by washing and addition of 20 µM H_2_DCFDA in HBSS (with Ca^2+^) for 45 min before being read at Ex_485 nm_ and Em_530 nm_ with a fluorescence microplate reader (Molecular Devices, Sunnyvale, CA). The results were presented as the ratio of the fluorescence of gp120–treated samples to that of the control samples.

### Superoxide assay

The production of O_2_^-^ by human microglial cultures treated with gp120 was determined by measuring the superoxide dismutase (SOD)-inhibitable reduction of ferricytochrome *c*, as previously described [130] with slight modification. Human mesencephalic neuronal/glial cultures (1x10^5^ cells/well) were incubated with gp120 (0.4 to 10 nM) for 3 days followed by washing and addition of HBSS with or without SOD (500 U/ml) and then ferricytochrome *c*. Plate was read at 550 nm with a microplate reader.

### Microglial cell migration assay

Highly purified human microglia were added to upper chambers of a 96-well chemotaxis device separated from the lower chambers with an 8µm pore size of polyvinylpyrrolidone-free polycarbonate filter. The lower chambers were filled with supernatants from the human mesencephalic neuronal/glial cultures. After 3 h of incubation, human microglia that had migrated from upper chambers into lower chambers were quantified by Diff-Quik staining. 

### Enzyme-linked immunosorbent assay (ELISA)

Supernatants of cell cultures after treatment will be harvested for ELISA analysis as previously described [50]. Briefly, ELISA plates (96-well) will be coated with mouse anti-human IL-1β, CCL2, CXCL10 or CX3CL1 antibodies overnight at 4°C. The plates will be blocked with 1% BSA in PBS for 1h at 37°C. After washing with PBS with Tween 20, culture supernatants and a series of dilutions of IL-1β, CCL2, CXCL10, or CX3CL1 (as standards) will be added to wells for 2h at 37°C. Following washing, detection antibody (goat anti-human IL-1β, CCL2, CXCL10 or CX3CL1 antibodies) will be added for 90 min at 37°C followed by donkey-anti-goat IgG horseradish peroxidase conjugate (1:10,000) for 45 min. A chromogen substrate K-Blue will then be added at room temperature for color development which will be stopped with 1M H_2_SO_4_. The plates will be read at 450 nm to generate standard concentration curves for IL-1β, CCL2, CXCL10 or CX3CL1 concentration extrapolation.

### Statistical analysis for functional assays

For comparison of the means of two groups, Student’s *t*-test was used. For comparison of means of multiple groups, analysis of variance (ANOVA) was performed followed by either Fisher’s protected least significant difference (PLSD)-test or Scheffe-test.
